# Predicting Work–Family Balance: A New Perspective on Person–Environment Fit

**DOI:** 10.3389/fpsyg.2019.01804

**Published:** 2019-08-06

**Authors:** Pei Liu, XiaoTian Wang, Aimei Li, Lei Zhou

**Affiliations:** ^1^School of Management, Jinan University, Guangzhou, China; ^2^School of Business Administration, Guangdong University of Finance and Economics, Guangzhou, China

**Keywords:** work–family integration, work–family balance, person–environment fit, family functioning, marital satisfaction, polynomial regression

## Abstract

Work–family balance continues to be a burgeoning topic of organizational research, yet, while the various antecedents of work–family balance have been identified, researchers have, to date, neglected the effect of congruence versus incongruence with regard to work–family integration preferences and the corresponding supplies at work. The current research investigates whether work–family integration preferences and organizational supplies jointly affect work–family balance, and the distal family–related outcomes including marital satisfaction and family functioning, from a person–environment fit perspective. Polynomial regression analysis and response surface methodology are used to test the study’s hypotheses. Results of the polynomial regressions on 393 employees are found to support the congruence effect hypotheses. In particular, the results show that employee work–family balance is higher when work–family integration preferences and organizational supplies are congruent, as opposed to incongruent. An individual’s balance is higher when preferences and supplies are aligned at higher levels rather than at lower levels. In addition, the asymmetrical shape of the surface along the incongruence line indicated that an employee’s work–family balance tends to be damaged once organizational supplies exceed personal preferences. Moreover, through creating a block variable based on the five polynomial terms, we found that congruence/incongruence in respect of work–family integration preferences and supplies yields distal effects on both family functioning and marital satisfaction. Our findings support our hypotheses and are also in line with both person–environment fit theory and balance theory. Theoretical and practical implications for keeping work-family balance are also discussed.

## Introduction

An increasing number of employees, especially those who are in a dual-earner couple relationship, are confronted with the challenge of making decisions regarding how to allocate their time and energy with respect to their work and family roles. Meanwhile, the operation of egalitarian social norms has altered the traditional family structure advocating for men as breadwinners and women as homemakers (Trent and South, 1992). A considerable body of literature has emerged to focus on the effect of such changes at the work and family interface, moving from a work–family conflict perspective to a work–family enrichment standpoint (Frone, 2003; Carlson et al., 2010; Barber et al., 2017). Correspondingly, the phenomenon of work–family balance has attracted considerable research attention (Valcour, 2007; Greenhaus and Allen, 2011). Despite the popularity of the construct, though, only recently have scholars conducted empirical studies in relation to it. Moreover, “work–family balance” varies in terms of its definition. Early studies defined it as the simultaneous experience of low conflict and high enrichment (Frone, 2003; Casper et al., 2014). Other, more recent researchers have proposed that work–family balance is distinct from conflict and enrichment, instead considering balance as a global assessment of the interplay between work and family (Valcour, 2007; Greenhaus and Allen, 2011). A commonly used evaluation of “balance” is balance satisfaction, representing individuals’ affective responses to the fit and allocation of time and attention between work and family (Valcour, 2007). Prior literature has documented a variety of influence factors in respect of work–family balance, including work-related variables such as job demands (Beham and Drobnič, 2010), personality types such as proactive personality (Aryee et al., 2005), and family-related factors such as income (Lu et al., 2009).

In addition, work–family boundary dynamics have also received increasing attention in the work and family domains. “Work–family boundary dynamics” concern the socially constructed lines between work and family domains, and how people maintain, negotiate, and transition these lines. Boundary theory can be used to understand individuals’ boundary management. According to this theory, there are cognitive, physical, and behavioral boundaries between work and family domains (Ashforth et al., 2000), and these boundaries comprise continuums, moving from sharpened points at which role segmentation occurs to blurred ends that lead to role integration. Different people have different preferences for work and family integration or segmentation. Research on individuals’ work–family boundary preferences has examined its effect on work–family conflict, suggesting that a high level of work–family segmentation preferences reduces work-to-family conflict (Powell and Greenhaus, 2010). Apart from individuals’ preferences, workplaces also vary in their practices or policies to manage distinct work and home boundaries (Kreiner, 2006). It is important to consider organizational policies and practices regarding work–family boundaries and individuals’ boundary preferences simultaneously, as congruence in organizational supplies and employee preferences may contribute to reductions in work–family conflict and increases in work–family enrichment. Accordingly, using the person–environment (hereafter, “P–E”) fit approach (Edwards, 2008) to examine congruence/incongruence in work–family boundaries may provide us with a more comprehensive way to understand the phenomenon of work–family balance.

Despite a considerable amount of research on work–home boundary and work–family balance, several gaps exist in the current literature. First, although two prior studies have examined the effect of the work–family boundary on work–family conflict and enrichment from a P–E fit perspective (Kreiner, 2006; Chen et al., 2009), no researchers have yet focused on the effect of congruence in work–family boundaries on work–family balance, specifically considering that balance is a different concept from conflict and enrichment (Wayne et al., 2017). Second, the studies conducted by Chen et al. (2009) and Kreiner (2006) only focused on the proximal effects of congruence in the work–family boundary on the work–family phenomenon (i.e., conflict and enrichment), overlooking its distal effects on family outcomes. Testing for specific family outcomes is important because it can extend our understanding of the role of congruence in the work–family boundary in relation to the family domain. Third, existing literature on P–E incongruence focuses mainly on how outcomes vary based on two different types of incongruence; that is, consequences based on the condition that the levels of person-related variables are higher than the levels of situation-related variables or outcomes brought about by the condition that the levels of person-related variables are lower than the levels of situation-related variables have been explored. However, no prior research has compared the two different types of incongruence, which may be significant in terms of understanding extensively the effects of P–E misfit (Edwards and Rothbard, 1999).

To address these research gaps, the current research, based on a P–E fit perspective, explores both the proximal effect (i.e., work–family balance) and distal effects (i.e., see section “Marital Satisfaction” and “Family Functioning”) of P–E congruence/incongruence in the work–family boundary. Moreover, we explore the influence of work–family balance through a P–E fit theoretical lens coupled with two advanced methodologies: response surface methodology and a mediation test through creating a “block” variable. Notably, response surface methodology allows us to test the interacting effect of personal preferences for work–family boundary management and organizational supplies in work–family boundary management in predicting work–family balance. Response surface methodology is more accurate than other techniques when testing P–E fit on outcomes (Edwards, 2002). Meanwhile, creating a new *block* variable when testing the mediating effect provides us with a way to examine the effect of P–E fit on important distal outcomes in the family domain. In addition, we also compare two different types of incongruence and examine their influences on work–family balance.

## Theory and Hypotheses

### Work–Family Balance

As previously noted, there are various definitions of “work–family balance.” Early researchers considered it to be the absence of work–family conflict (Buffardi et al., 1999). However, an increasing number of subsequent studies have contended that this absence cannot embody work–family balance in its entirety. For instance, Frone (2003) proposed that work–family balance is a simultaneous perception of low work–family conflict and high work–family enrichment. This kind of definition argues that work–family balance includes both negative and positive inter-role experiences, and has been the most common conceptual definition used in related studies (Casper et al., 2014). Researchers who use this definition typically measure both work–family conflict and work–family enrichment. For instance, Aryee et al. (2005) used both to represent work–family balance in their examination of the antecedents of balance, finding that four different dimensions of balance could be predicted by different antecedents. Given that such a definition is articulated through the combination of both conflict and enrichment, it is considered a combined “spillover” approach (Wayne et al., 2017).

Alternatively, other researchers have argued that distinguishing work–family balance into four dimensions is problematic because it becomes difficult to clarify the effect of work–family balance if both cross-domain processes and directions are taken into consideration. For instance, Aryee et al. (2005) found different causes and consequences were associated with work–family balance measured by both conflict (work–to–family conflict and family–to–work conflict) and enrichment (work-to-family enrichment and family-to-work enrichment). In which case, how might we draw conclusions about the antecedents and outcomes of work–family balance? Some scholars have proposed that balance is different from work–family conflict and enrichment, and that it should be regarded as a global construct that captures gestalt perceptions of the interactions between work and family roles (Voydanoff, 2005; Grzywacz and Carlson, 2007). Inclusive approaches such as this assess individuals’ overall perceptions after combining their work and family roles. Herein, a common indicator of work–family balance is “balance satisfaction,” which refers to an attitude to work and family roles representing the personal judgment of fit between resources and demands across work and family domains (Valcour, 2007). Similar to other attitudes, balance satisfaction involves cognitive components such as resource allocation, fit, and integration across work and family roles, and emotional components such as emotional states. Balance satisfaction focuses on personal thoughts and emotions about work and family balance, and, as such, is a psychological construct based on internal and subjective assessment. Moreover, it differs from combining job satisfaction and family satisfaction separately because it is an integrated concept across work and family roles (Carlson et al., 2013). Wayne et al. (2017) compared the additive spillover approach and the global evaluation approach and found that the former was the most important predictor of work outcomes (e.g., organizational commitment and job satisfaction), whereas the latter (i.e., balance satisfaction) was a significant predictor of family-related outcomes. Given that the current research is concerned with the distal effects of congruence/incongruence in the work–home boundary on family outcomes, we selected balance satisfaction as the indicator of work–family balance in our study.

Additionally, extant studies have tended to examine the causes of balance satisfaction in relation to either work–related characteristics or family–related attributes. For example, Abendroth and den Dulk (2011) found that work pressure negatively predicted balance satisfaction, while household task harmony was positively related to it. However, no prior research has focused on the interacting effect of the person and the environment in predicting balance satisfaction. Therefore, in the present study, we examine how congruence/incongruence in the work–family boundary affects work–family balance satisfaction from a P–E fit perspective.

### P–E Fit

“P–E fit” theory proposes that, even though a person and an environment can affect some outcomes separately, the interaction between the two is more important to consider. There are two common versions of P–E fit theory: supplies–values fit and demands–abilities fit. The former is the fit of personal motives, goals, and interests with the supplies provided by the environment. Here, “supplies” refers to rewards in the environment, and encompasses external rewards (e.g., pay) as well as those resulting from the individual’s experiences in the environment. Demands–abilities fit is the match between an individual’s abilities and the requirements of the organization. “Requirements” in the organizational context involve objective demands, such as work time and socially constructed norms, and subjective demands, such as role expectations in the workplace. The P–E fit approach has been widely used in the workplace and organizational environment to test the relationships between P–E fit and work-related variables, such as job satisfaction (e.g., Edwards and Rothbard, 1999), and person–related variables, such as strain and well–being (e.g., Yang et al., 2008).

Many studies exploring the relationship between P–E fit and outcomes (e.g., well-being) rely on two simple hypotheses. The first is that outcomes may be maximized or minimized when person and environment align at an optimum level. The second is that P–E fit results in the same levels of change in outcomes regardless of the absolute levels of person-related and situation-related variables. That is, outcomes may endure at the same level when person-related variables and situation–related variables are aligned at either a high level or low level. However, such assumptions fail to address more complex options, such as whether the P–E fit aligned at a low level is the same as the fit aligned at a high level. Edwards and Rothbard (1999) found that P–E fit and outcomes follow a variety of functional forms. To capture the multilayered relationships between P–E fit and outcomes, three basic questions corresponding to the fundamental attributes of person-related variables and situation-related variables were posed as follows: (1) Do outcomes improve, reduce, or remain constant as levels of situational variables increase toward levels of person-related variables? (2) How do outcomes change when levels of situational variables exceed levels of person-related variables? (3) Do outcomes remain constant regardless of the aligned levels (high vs. low) between situation–related variables and individual variables? Obtaining answers to these questions should help us better comprehend the complex relationship between P–E fit in work–family boundary and work–family balance. Additionally, we asked a further question: (4) Is work–family balance better or worse when an individual’s preferences regarding the work–family boundary are higher than organizational supplies compared to when personal preferences are lower than organizational supplies? Dealing with this question allows us to compare two issues corresponding to the incongruence line, which has received increasing attention in recent years (Zhang et al., 2012; Wilson et al., 2018). In the following sections, we focus on work–family integration, one end of the work–family boundary, and answer each question to discover the relation between P–E congruence in work–family integration and work–family balance. P–E fit theory and balance theory provide theoretical support for our hypotheses.

### P–E Fit and Work–Family Balance

According to P–E fit theory, work–family balance will increase when organizational integration increases toward an individual’s preferences, since sufficient supplies mean fulfilled needs, desires, and goals. Within this scenario, the individual and the organization have consistent goals, and employees perceive plenty of support from the organization (Edwards and Rothbard, 1999).

Balance theory proposes that individuals prefer to maintain a balanced or harmonious state in which entities that individuals possess and the feelings induced by such entities fit together without stress (Heider, 1958). “Entities” refer to anything individuals own and use, or subjects produced though individuals’ or others’ actions (Heider, 1958). Work–home integration is a kind of policy maintained by the organization, and is also an entity that an individual can use to deal with their work–family issues. The core ideas of balance theory are that people pursue balanced states, while imbalance yields negative feelings and a pressure for change. A P–E fit in respect of work–home integration represents a balanced state wherein individuals’ integration preferences can be fulfilled by supplies of workplace integration. Such congruence enables employees to cope with their work and family demands in a similar fashion as their organizational culture. For example, if the organization also advocates high integration between work and family, an employee with high levels of integration preferences considers that his or her goals can be met easily. Individuals may also perceive they are justified in their own efforts within the work and family domains, given that the workplace has similar policies or cultures in meeting both work and family demands. Moreover, the congruence in work–family integration indicates that an individual’s own resource allocation decisions toward work and family are validated by their organization, promoting a balanced perception of work and family (Grawitch et al., 2013). However, incongruence in work–home integration results in an imbalanced state in which individuals experience great tension. As a result, an individual may question the effectiveness of their resource allocation between work and family, diminishing their balance satisfaction (Valcour, 2007). Furthermore, if a misfit of work–home integration occurs, individuals can perceive low support derived from their organizations, resulting in an increase in perceptions of inter-role conflict (e.g., Byron, 2005). Thus, individuals will experience lower balance satisfaction. Taking these points together, we propose:

Hypothesis 1: The more aligned an employee’s work–family integration preferences and workplace supplies are, the higher their level of work–family balance.

Incongruence in work–family integration can be seen to take two different forms. In the present study, we focus on changes in an individual’s work–family balance as a misfit moving from organizational supplies having higher levels of work–family integration than personal preferences to personal preferences having higher levels of work–family integration than organizational supplies. Edwards (1996) posited that four different processes occur when supplies exceed an individual’s values: depletion, interference, conservation, and carryover. In “depletion,” excess supplies make one’s values on the same dimension less likely to be met in the future. “Interference” occurs when one’s supplies–values fit in other dimensions is inhibited because of excessive supplies. “Conservation” is the opposite side of depletion, whereby excess supplies are conserved to meet values on the same dimensions in the future. Similarly, “carryover,” in contrast to interference, means that excess supplies can be used to fulfill values on other dimensions. The former two processes yield inferior outcomes, whereas the latter two yield enhanced outcomes.

In the present research, we expect that interference is more likely to occur when organizational supplies exceed personal preferences for work–family integration. A person may experience great pressure when their organization excessively advocates to integrate work and family roles (Kreiner, 2006). Individuals may need to handle a lot of work when they stay at home because of policies and practices driven by their workplace. Thus, they will likely use a large amount of their limited time and yet still struggle to cope with their work, diminishing their balance satisfaction because they do not have enough time and energy to help them meet their family responsibilities (Ferguson et al., 2015). Conversely, even though incomplete supplies in the workplace can produce an imbalanced state, individuals are prone to reallocating their resources to fulfill their own preferences. For instance, if the workplace allows employees to segment their work and family roles (low integration), employees who have high levels of integration preferences are more willing to spend their time at home dealing with their work, resulting in higher balance satisfaction. Empirical evidence has found that excess supplies increases individuals’ work–family conflict and stress (Kreiner, 2006). Taking these theoretical and empirical perspectives together, we propose:

Hypothesis 2: Employee work–family balance is lower when organizational supplies in work–home integration exceed personal preferences, compared to when personal preferences exceed organizational supplies.

Congruence in work–family integration also has different dimensions. In this study, we are interested in how individuals’ work–family balance varies when both supplies and values are aligned at high levels or low levels. We expect that work–family balance will remain constantly high when supplies and values are perfectly aligned (at high or at low levels) because an employee can achieve his or her goals regarding the integration of work and family. When work–home integration supplies provided by the workplace and personal integration preferences are both high, individuals obtain support derived from their organizations, which consequently justifies individuals’ resource allocation between work and family (Ferguson et al., 2015; Kreiner, 2006). In addition, differing from the findings of previous studies that have suggested well-being or satisfaction will decrease when values and supplies are both at low levels versus high levels (Edwards and Rothbard, 1999), we posit that individuals’ balance satisfaction will not decrease in such scenarios because their desires and needs can also be fulfilled by similar organizational supplies. Existing literature has suggested that individuals’ work–family conflict is kept constant irrespective of whether organizational supplies of work–home segmentation and personal preferences are aligned at high levels or low levels (Kreiner, 2006), providing indirect evidence for our assumptions given that some researchers have proposed that work–family balance occurs when work–family conflict is absent. Taking these viewpoints together, we propose:

Hypothesis 3: Employees’ work–family balance remains constant no matter whether their work–family preferences and workplace supplies are congruent at high or low levels.

### Work–Family Balance and Family Outcomes

Prior research has conceptually stated and empirically shown positive relationships between work–family balance and family outcomes, with most studies operationalizing work–family balance in terms of low work–family conflict and high work–family enrichment. Results from existing literature suggest that work–family balance positively predicts marital satisfaction (Allen et al., 2000), family performance, and time allocation in domestic activities (Clarke et al., 2004). Even in the few studies examining the relationship between work–family balance and family outcomes through the lens of balance as a global concept, similar results have been found. For instance, Grawitch et al. (2013) reported a positive association between balance satisfaction and non–work satisfaction. Wilson et al. (2018) found that greater balance satisfaction was related to greater relationship satisfaction. Given that balance satisfaction means a balanced state in which individuals feel they have sufficient resources with which to cope with demands derived from work and family domains (Valcour, 2007), it is reasonable to infer that individuals with greater balance satisfaction will be more prone to experiencing positive outcomes in their family, such as enhanced marital satisfaction, a global evaluation of the extent of happiness married couples experience in their relationship (Glenn, 1990), as well as family functioning, a principal aspect of which concerns the variety of interactions among family members as well as the interactions with social systems beyond home domains (Miller et al., 2000).

We have hypothesized the effect of P–E fit in work–family integration on work–family balance and the positive relationship between work–family balance and family outcomes (i.e., see section “Marital Satisfaction” and “Family Functioning”), and we further expect that work–family balance carries these P–E fit effects into an employee’s marital satisfaction and family functioning. That is, we hypothesize a mediating role for work–family balance, suggesting that P–E fit in work–family integration is important to the family domain because it affects marital satisfaction and family functioning via employees’ enhanced work–family balance:

Hypothesis 4: Work–family balance mediates the relationship between P–E fit in work–family integration and employees’ (a) family functioning and (b) marital satisfaction.

## Materials and Methods

### Participants and Procedure

A convenience sample of working adults from seven Chinese organizations (i.e., three securities companies, two business consulting companies, and two software companies) was recruited to participant in the current study. To be included in the study, participants had to work a traditional work week and be married. All surveys were completed via wjx.com, a professional questionnaire survey site in China. Before starting the formal survey, participants were told the purpose of this research, the voluntary nature of their participation, and the confidentiality and anonymity of their responses. After they consented to participate in the study, the interface associated with our questionnaires was presented. In addition, we set a minimum time (3 min) to complete each survey to control the authenticity of participants’ responds. We also restrict the same IP address in each survey to only complete questionnaire once to avoid multiple questionnaires being completed by the same individual. Numerous prior studies have used the WJX platform to survey Chinese participants as it allows researchers to set specific qualifications to ensure the quality of data (e.g., Wang et al., 2018; Liu et al., 2019).

Participants completed two surveys at two different time points within 1 month. They first received a link to a survey that included questions regarding demographics, work–family integration preferences, and organizational supplies questions. The next survey, including work–family balance, marital satisfaction, and family function questions, was administered 1 month later. Of 500 employees contacted for the first survey, 393 people completed the surveys at both time points, resulting in a response rate of 78.6%. In terms of demographic characteristics, the majority of the participants were male (51.4%) and 31 to 40 years old (62.1%); 296 employees had one child (75.3%), and 189 participants (48.1%) had worked at their organization for between five and 10 years. Among those participants, 175 (44.5%) were first-line managers. The average number of working hours per week was 43.59 and the average commuting time per day was 73.24 min.

### Measures

We used Brislin’s (1980) translation/back–translation procedure to translate the English versions of all questionnaires into Chinese. Initially, the first author, who is fluent in both English and Chinese, translated the questionnaires into Chinese. Then, the corresponding author, who is also fluent in both languages, translated the Chinese versions back into English. Finally, the two English versions, including all questions and integrated subscales (see below), were compared to check whether there were any inconsistencies. Some minor discrepancies were resolved via further discussions among the authors.

#### Work–Family Integration Preferences and Organizational Supplies

We adapted two 4-item scales developed by Kreiner (2006) to assess employees’ work–family integration preferences and organizational supplies at Time 1. The measure of employees’ work–family integration preferences reflected the degree to which individuals prefer to integrate their work life and family life, while the measure of organizational supplies reflected individuals’ perception of the organization supporting (or not) their preferences for integration. Responses ranged from 1 (*strongly disagree*) to 7 (*strongly agree*). A sample preference item is “I like work issues creeping into my home life.” A corresponding sample workplace item is “At my workplace, people allow work issues to creep into their home lives.” Cronbach’s alpha for integration preference items in this study was 0.92, and for organizational supplies was 0.85.

#### Work–Family Balance

Satisfaction with work–family balance was measured using a four-item scale developed by Valcour (2007) at Time 2. An example item is “Are you satisfied or dissatisfied with the way you divide your attention between work and home?” Responses were scored on a 5-point Likert scale ranging from 1 (*very dissatisfied*) to 5 (*very satisfied*). Cronbach’s alpha was 0.81.

#### Family Functioning

Six items from the “family assessment device” (Epstein et al., 1983) were used to assess employees’ family functioning at Time 2. Each item was rated on a 5-point Likert scale ranging from 1 (*strongly agree*) to 5 (*strongly disagree*). A sample item is “We are able to make decisions about how to solve problems.” Cronbach’s alpha was 0.70.

#### Marital Satisfaction

We measured marital satisfaction by using the nine-item scale from the Chinese version of the ENRICH marital inventory (Jiang et al., 2015) at Time 2. Items were presented in a 5-point Likert scale ranging from 1 (*strongly disagree*) to 5 (*strongly agree*). Example items are “I am very happy with how we handle role responsibilities in our marriage” and “I am not happy about our communication and feel my partner does not understand me” (reversed score). The coefficient alpha was 0.81.

#### Control Variables

Previous researchers have found that gender, work hours, and commuting time are related to work–family balance (Voydanoff, 2005; Valcour, 2007; Wilson et al., 2018). Additionally, the relationship between age and marital satisfaction is already well established (Guilford and Bengtson, 1979). The number of children an employee has is also thought to be related to work–family balance satisfaction, as more children can mean an increase in the difficulty of meeting work and family demands, which then is negatively associated with work–family balance satisfaction (Valcour, 2007). Therefore, we assessed and controlled for participant gender (1 = male, 2 = female), age (1 = 18–25 years old, 2 = 26–30 years old, 3 = 31–40 years old, 4 = 41–50 years old, 5 = 51–60 years old, and 6 = more than 60 years old), organizational tenure (1 = less than 6 months, 2 = 1–3 years, 3 = 3–5 years, 4 = 5–10 years, 5 = more than 10 years), number of children (1 = no children, 2 = one child, 3 = 2 children, 4 = more than 2 children), work hours (average hours worked per week), and commuting time (average commuting time per day).

### Data Analysis

#### Polynomial Regressions

To test the congruence and asymmetrical incongruence effects of individuals’ work–family integration preferences and organizational supplies on work–family balance satisfaction and family outcomes (i.e., see section “Family Functioning” and “Marital Satisfaction”), polynomial regressions and response surface modeling were used (Edwards and Parry, 1993; Jansen and Kristof-Brown, 2005). Polynomial regressions can generate three-dimensional response surfaces, uncovering the congruence effects on outcome variables. In the current research, work–family balance was regressed on five polynomial terms, as follows: employee work–family integration preferences (*P*), organizational supplies in work–family integration (*S*), employee work–family integration preferences squared (*P*^2^), organizational supplies in work–family integration squared (*S*^2^), employee work–family integration preferences multiplied by organizational supplies in work–family integration (*P* × *S*). The specific formula was as follows:

Z=b+0bP1+bS2+bP3+2b(P×S)4+bS5+2e

In addition, *P* and *S* were centered around the pooled grand mean before calculating the second-order terms, the purpose of which was to reduce multicollinearity.

The slopes and curvatures along both the congruence line (*P* = *S*) and the incongruence line (*P* = –*S*) were examined following completion of the polynomial regressions procedure. The results that the three second–order polynomial terms (i.e., *P*^2^, *P* × *S*, and *S*^2^) were jointly significant and the curvature along the incongruence line differed significantly from zero can confirm a significant congruence effect (Hypothesis 1). Additional tests were also conducted to examine whether the surface along the incongruence line was symmetric (Hypothesis 2). The symmetry of the surface along the incongruence line depends on a lateral shift of the response surface along the incongruence line, the formula for which is (*b*_2_ − *b*_1_)/2 × (*b*_3_ − *b*_4_ + *b*_5_) (Atwater et al., 1998). A significant positive lateral shift as well as negative curvature denotes that outcomes are lower in the region where *P* < *S* along the incongruence line, while higher levels of outcomes are found in the same region when both significantly negative values of lateral shift and curvature are reported. Moreover, a non-significant estimated slope value along the congruence line suggests that congruence at high levels of predictors do not result in significantly higher or lower outcomes, compared to congruence at low levels of predictors (Hypothesis 3).

#### Mediation Test

To test the mediating effect of work–family balance satisfaction in the relationships between individual work–family integration preferences and organizational supplies congruence/incongruence and family outcomes, the block approach was used. Specifically, the five polynomial terms were combined to form a new block variable. The weights were the estimated coefficients in the original polynomial regressions. The polynomial regressions were rerun by using the block variable and the unstandardized regression coefficient for the block variable was obtained. The mediating effect was examined through two models. One tested the predicting effect of the five polynomial terms on the work–family balance satisfaction. The other model added family outcomes to the regression to examine the effect of family outcomes after controlling for the congruence/incongruence effects. In addition, the significance of indirect effects was tested through bootstrapping 20,000 samples with bias-corrected estimation, conducted using *M*plus 8.1 (Muthén and Muthén, 2017). One of the major advantages of using the block variable approach when testing indirect effect is that this approach does not change the original estimated coefficients for other variables in the equation, nor the total explained variances.

## Results

Means, standard deviations, and intercorrelations among all variables are presented in Table 1. As shown in Table 1, work–family integration preferences were positively correlated with work–family balance (*r* = 0.14, *p* < 0.01). Employee perception of organizational supplies in work–family integration was negatively correlated with work–family balance (*r* = –0.43, *p* < 0.01), family functioning (*r* = –0.16, *p* < 0.01), and marital satisfaction (*r* = –0.17, *p* < 0.01). Work–family balance was positively correlated with both family functioning (*r* = 0.32, *p* < 0.01) and marital satisfaction (*r* = 0.38, *p* < 0.01). Moreover, family functioning was positively correlated with marital satisfaction (*r* = 0.65, *p* < 0.01).

**TABLE 1:** Mean, deviations, and correlations for all variables.

	**1**	**2**	**3**	**4**	**5**	**6**	**7**	**8**	**9**	**10**	**11**
(1) Gender (1 = male)	—										
(2) Age	0.04	—									
(3) Organizational tenure	0.04	0.63^∗∗^	—								
(4) Number of children	–0.04	0.18^∗∗^	0.07	—							
(5) Work hours per week	–0.20^∗∗^	–0.03	0.05	–0.02	—						
(6) Commuting time per day	–0.07	0.00	0.02	–0.02	0.01	—					
(7) Personal preferences for work–family integration	−0.10^∗^	0.05	–0.02	0.03	–0.01	–0.04	(0.92)				
(8) Organizational supplies in work–family integration	–0.07	–0.02	–0.01	0.01	0.23^∗∗^	0.05	0.02	(0.85)			
(9) Work–family balance	–0.05	0.01	0.002	0.01	−0.11^∗^	0.07	0.14^∗^	–0.43^∗∗^	(0.81)		
(10) Family functioning	0.02	–0.01	0.03	–0.04	0.05	0.01	–0.03	–0.16^∗∗^	0.32^∗∗^	(0.70)	
(11) Marital satisfaction	–0.07	–0.01	–0.01	–0.01	–0.01	0.02	0.00	–0.17^∗∗^	0.38^∗∗^	0.65^∗∗^	(0.81)
*M*	1.49	2.81	4.99	2.10	43.59	73.24	3.11	3.66	3.76	4.30	3.96
*SD*	0.50	0.73	0.87	0.50	10.87	87.28	1.63	1.35	0.78	0.44	0.59

**N* = 393. Reliability coefficients are reported along the diagonal. ^∗^*p* < 0.05. ^∗∗^*p* < 0.01.*

To examine the distinctiveness of the five self-reported variables (i.e., see section “Work–Family Integration Preferences and Organizational Supplies,” “Work–Family Balance,” “Family Functioning,” and “Marital Satisfaction”), confirmatory factor analysis was conducted using *M*plus 8.1. Results showed that the five-factor model was acceptable, χ^2^(314) = 593.97, *p* < 0.001, CFI = 0.93, TLI = 0.92, RMSEA = 0.05, SRMR = 0.05. Another six additional models were also tested (see Table 2). Models 2 to 6 loaded two or three latent variables on a common factor, and Model 7 was a single-factor model. Table 2 presents the results of model fit comparisons, which indicated that our hypothesized model was significantly better than any of the alternative models.

**TABLE 2:** Model fit results for confirmatory factor analysis.

**Model**	**χ^2^**	***df***	**CFI**	**TLI**	**RMSEA**	**SRMR**	**Δχ^2^(Δ*df*)**
(1) Hypothesized five-factor model	593.97^∗∗∗^	314	0.93	0.92	0.05	0.05	
(2) Model 2: Personal preferences and organizational supplies were combined	1744.08^∗∗∗^	318	0.64	0.61	0.11	0.10	1150.11^∗⁣∗∗^(4)
(3) Model 3: Family functioning and marital satisfaction were combined	618.42^∗∗∗^	318	0.93	0.92	0.05	0.05	24.45^∗⁣∗∗^(4)
(4) Model 4: Work–family balance and family functioning were combined	929.40^∗∗∗^	318	0.85	0.83	0.07	0.08	335.43^∗⁣∗∗^(4)
(5) Model 5: Work–family balance and marital satisfaction were combined	1019.29^∗∗∗^	318	0.83	0.81	0.08	0.08	425.32^∗⁣∗∗^(4)
(6) Model 6: Work–family balance, family functioning, and marital satisfaction were combined	1055.30^∗∗∗^	321	0.82	0.80	0.08	0.08	461.33^∗⁣∗∗^(7)
(7) Model 7: Single-factor model	2775.50^∗∗∗^	324	0.39	0.34	0.14	0.13	2181.53^∗⁣∗∗^(10)

**N* = 393. *df*, degree of freedom; CFI, comparative fit index; TLI, Tucker–Lewis index. RMSEA, root mean square error of approximation; SRMR, standardized root mean square residual. Δχ^*2*^ were obtained by comparison with Model 1. All alternative models were compared with the hypothesized model. ^∗∗∗^*p* < 0.001.*

**FIGURE 1:**
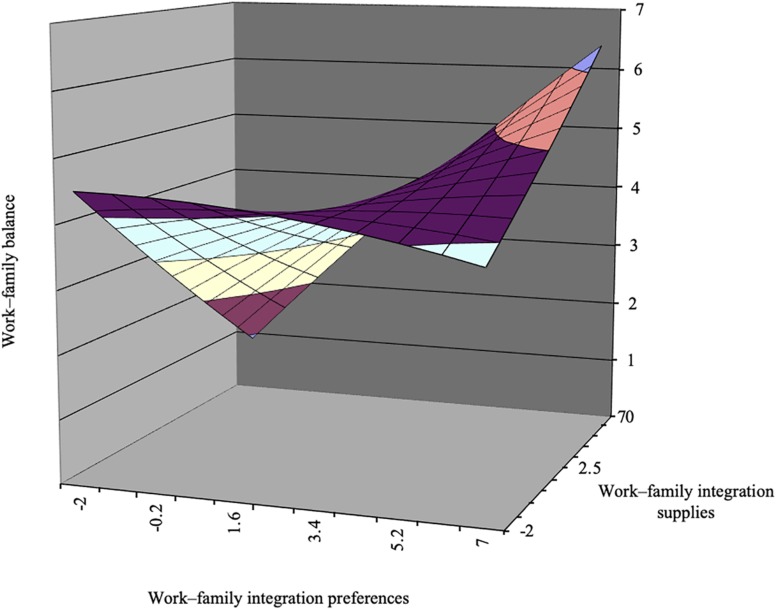
Congruence effect and asymmetrical incongruence effect of personal preferences for work–family integration and organizational supplies in work–family integration on work–family balance satisfaction.

Hypothesis 1 suggested that there would be a congruence of individual preferences and organizational supplies on work–family balance. The results of the estimated coefficients of first-order and second-order terms, as well as the slopes and curvatures along both the congruence and incongruence lines for the cross-level polynomial regressions, are shown in Table 3. Figure 1 illustrates the response surface based on these coefficients. The results reveal that the three second-order terms were jointly significant, *F* = 9.99, *p* < 0.01. The curvature along the incongruence line was negative (–0.08, *p* < 0.01). As shown in Figure 1, the surface is downward, indicating it is an inverted U-shaped one along the incongruence line. The congruence line is from the left corner (*P* = *S* = –2) to the right corner (*P* = *S* = 7). The line perpendicular to the congruence line is the incongruence line. The negative curvature along the *P* = –*S* line indicates that work–family balance was higher when individual preferences were aligned with organizational supplies, and any deviation from the congruence line decreased balance satisfaction, thus supporting Hypothesis 1.

**TABLE 3:** Cross-level polynomial regression results and path analysis results.

**Variables**	**Balance satisfaction**	**Family functioning**		**Marital satisfaction**	
		**Model 1**	**Model 2**	**Model 1**	**Model 2**
Constant	3.94^∗∗^	4.06^∗∗^	3.27^∗∗^	4.03^∗∗^	2.75^∗∗^
Gender	–0.11	0.01	0.03	–0.10	–0.07
Age	–0.02	0.00	0.004	0.02	0.02
Organizational tenure	0.02	–0.004	–0.008	–0.04	–0.04
Number of children	0.01	–0.03	–0.03	–0.01	–0.01
Work hours per week	–0.003	0.004	0.004	0.001	0.002
Commuting time	0.001	0.00	0.00	0.00	0.00
Personal preferences for work–family integration (*P*)	0.11^∗∗^	–0.08^∗∗^	–0.10^∗∗^	–0.08^∗∗^	–0.12^∗∗^
Organizational supplies in work–family integration (*S*)	–0.26^∗∗^	–0.03	0.02	−0.05^∗^	0.03
*P*^2^ (*b*_3_)	–0.01	0.05^∗∗^	0.05^∗∗^	0.05^∗∗^	0.06^∗∗^
*P* × *S* (*b*_4_)	0.08^∗∗^	0.01	–0.004	0.001	−0.02^∗^
*S*^2^ (*b*_5_)	0.003	0.003	0.002	0.01	0.01
Work–family balance			0.20^∗∗^		0.33^∗∗^
*R*^2^	0.28		0.20		0.20
Δ*R*^2^	0.06		0.08		0.13
Congruence (*P* = *S*) line					
Slope (*b*_1_ + *b*_2_)	–0.15^∗∗^		–0.10^∗∗^		–0.12^∗∗^
Curvature (*b*_3_ + *b*_4_ + *b*_5_)	0.07^∗∗^		0.07^∗∗^		0.06^∗∗^
Incongruence (*P* = –*S*) line					
Slope (*b*_1_ − *b*_2_)	0.38^∗∗^		–0.06		–0.03
Curvature (*b*_3_ − *b*_4_ + *b*_5_)	–0.08^∗∗^		0.04^∗^		0.06^∗∗^
*F* for the three quadratic terms	9.99^∗∗^		12.38^∗∗^		11.18^∗∗^

*^∗^*p* < 0.05. ^∗∗^*p* < 0.01.*

To test Hypothesis 2, the lateral shift value was calculated. The value was 2.27, indicating that work–family balance was lower in the region where personal preferences were lower than organizational supplies. Thus, Hypothesis 2 was supported.

To test Hypothesis 3, the significance of the slope along the congruence line was determined. As shown in Table 3, a significant slope along the congruence line (-0.15, *p* < 0.01) was found, indicating that employees’ work–family balance was higher when personal preferences and organizational supplies were aligned at a lower level than at a higher level. Thus, Hypothesis 3 was not confirmed.

To test the mediating effect of work–family balance in the relationships between personal preferences and organizational supplies congruence/incongruence and the various family outcomes, two polynomial regressions were run, as discussed above. The unstandardized coefficients are presented in Table 3. We found that the combined effect on work–family balance was significant, β = 0.52, *p* < 0.01. Moreover, balance positively predicted both family functioning, β = 0.20, *p* < 0.01, and marital satisfaction, β = 0.33, *p* < 0.01. The indirect effects of the block variable on family functioning and marital satisfaction were all significant, β = 0.18, *p* < 0.01, 95% CI [0.13, 0.25], and β = 0.22, *p* < 0.01, 95% CI [0.17, 0.29], respectively. In addition, neither the direct effect of the block variable on family functioning nor the direct effect of the block variable on marital satisfaction was significant, β = –0.04, *p* < 0.05, 95% CI [–0.16, 0.06], and β = –0.10, *p* > 0.05, 95% CI [–0.21, 0.002], respectively, indicating that work–family balance fully mediated the effects of work–family integration preferences and organizational supplies on family functioning and on marital satisfaction. Thus, Hypotheses 4a and 4b were both supported.

## Discussion

Despite the importance and the various family–related benefits of work–family balance (e.g., Grawitch et al., 2013), researchers have neglected to examine the congruence between individual work–home integration preferences and perceived levels of supplies at one’s workplace in affecting employee work–family balance and distal outcomes in the family domain. In the present research, we combined P–E fit theory and balance theory and found a beneficial effect of individual preferences and workplace supplies congruence on work–family balance and family outcomes. We also found that these effects of congruence on distal family outcomes were fully mediated by work–family balance in such a way that congruence was related to higher levels of balance, which in turn led to higher levels of family functioning and marital satisfaction. Furthermore, different patterns of incongruence in preferences and supplies resulted in different levels of negative effects on balance, which overcame the limitation of previous studies that overlooked the comparison between different patterns of P–E fit along the incongruence line. In particular, when individual preferences for work–family integration were lower than perceived supplies provided by the workplace, employee work–family balance satisfaction decreased more sharply than when individual preferences were higher than workplace supplies.

Regarding congruence, we expected that the levels of congruence in preferences and supplies would not lead to significant changes in work–family balance. However, this hypothesis (Hypothesis 3) was not supported. We found that employees’ work–family balance satisfaction was lower when preferences and supplies were congruent at higher levels versus at lower levels. Although the result was not consistent with our assumption, it is not to be discounted. First, compared to lower levels of work–family integration, higher levels of integrated work–family role boundaries often accompany cross-role interruptions and frequent time pressures, thereby increasing difficulties of meeting work and family demands (Chen et al., 2009). Thus, individuals are more likely to have difficulties in balancing their work and family responsibilities, reducing their subjective satisfaction with work–family balance. Second, some research on P–E fit in work–family segregation provides indirect evidence for our findings. Specifically, Edwards and Rothbard (1999) found that individuals’ well-being (i.e., with respect to anxiety and depression) was higher when value scores and supplies were congruent at higher levels versus lower levels. Similarly, Kreiner (2006) found that individuals perceived lower levels of work–family conflict if their work–home segregation preferences and organizational supplies were aligned at higher levels rather than at lower levels. Given that work–family boundaries are continuum, these results suggest that highly aligned work–family integration is more likely to have detrimental effects on outcomes.

### Theoretical Implications

The findings of the present research have several important theoretical implications. First, by integrating perceived supplies of integration at work as well as the P–E fit framework into a study of work–family boundary dynamics and the potentially associated outcomes in the family domain, we extend work–family boundary dynamics research by revealing that workplace supplies affect the likelihood for individual work–home integration preferences to translate into positive family outcomes. A wide variety of extant theoretical and empirical studies have proposed that individual work–home boundary preferences play a crucial role in family-related outcomes such as work–family enrichment (McNall et al., 2015) and family performance (Liao et al., 2016). However, these studies have overlooked contextual factors such as workplace supplies and the work–home boundary. Our results reveal that the real picture is far more complex and that the associated outcomes in the family domain depend on the congruence or incongruence at various levels of individual preferences and workplace supplies. In particular, the findings in the present study suggest that, depending on workplace supplies of work–family integration, individual preferences for work–family preferences may not always be beneficial or detrimental to family outcomes such as family functioning and marital satisfaction. Thus, the present study encourages us to incorporate contextual supplies at work into the theoretical framework for understanding the outcomes of work–family boundary dynamics.

Second, contributing to the work–family balance literature, the present study demonstrates that congruence/incongruence in individual work–home integration preferences and workplace supplies leads to different levels of work–family balance satisfaction. The asymmetrical incongruence effects in preferences and supplies we found suggest the importance of excessive workplace supplies in aggravating the potential negative effects of incongruence on satisfaction with work–family balance. We have shown that the mismatch between excessive workplace supplies of integration and lower levels of individual preferences will be more detrimental than the mismatch between lower levels of workplace supplies and higher levels of individual preferences. Overall, these complex patterns of effects suggest that work–family balance satisfaction varies based on the joint effects of individual work–home integration preferences and perceived supplies at work. These findings enrich existing theories toward a better understanding of how the effects of work–home boundary dynamics are carried through P–E relationships.

Third, the present study indicates that, contrary to our hypothesis and previous findings, work–family balance was lower when personal preferences and organizational supplies align at higher levels. High levels of organizational supplies of work–family integration suggests that organizational advocates and support can sustain work even during off-job time. High personal preferences indicate that an employee wishes to engage in work–related tasks at home (Kreiner, 2006). That is, the employee would like to allocate more finite resources such as time and energy to work–related activities during off–job time. However, due to the limited nature of those resources (e.g., time, self–regulatory resources), an employee who wants to allocate more resources at work during off–job time may not be able to do so (Ďuranová and Ohly, 2015). When congruence values of preferences and supplies are higher, prolonged work at home may hinder the fulfillment of employees’ family obligations through drawing on the finite resources, even though employees prefer to do so. Accordingly, these employees may find it’s difficult to accomplish work and family duties simultaneously and they will not perceive working during off–job time as being reasonable. Consequently, their work–family balance may decrease. For example, for highly ambitious employees, although supplemental working from home is preferred by them and encouraged by their organizations, they may not be able to balance work and family duties since the resources that are essential for both work and family activities are limited. Previous studies have provided indirect evidence for our inference. Hughes and Parkes (2007) found that higher work–time control buffered but did not diminished the effect of long work hours on work–family interference. Julien (2007) demonstrated that control over work-life interface didn’t moderate the relationship between job demands and work–life conflict.

Finally, this study found a fully mediating effect of work–family balance satisfaction in linking P–E fit in work–home integration to important family-related outcomes for employees. This integration of work–family balance satisfaction and P–E fit literatures offers additional insights into why different patterns of match and mismatch are associated with employees’ family functioning and marital satisfaction. Moreover, the present study extended the P–E fit literature by showing that the more nuanced effects of congruence levels and asymmetrical incongruence effects would be overlooked if we simply compare the effects caused by P–E congruence and incongruence. Examining different congruence and incongruence effects can offer additional theoretical insights for us to apply in understanding the nature of P–E fit, the importance of which has been noted in previous studies (e.g., Zhang et al., 2012; Wilson et al., 2018). For instance, Wilson et al. (2018) found that employee balance satisfaction was higher when their work–family conflict and their romantic partners’ work–family conflict was congruent at lower versus higher levels, while when an employee had a higher level of work–family conflict than his/her partner as compared to when the partner had a higher level of work–family conflict than the employee, employee balance satisfaction was lower. The results provided by Zhang et al. (2012) showed that leader-member exchange (LMX) quality would be higher when the leader’s proactive personality and the follower’s proactive personality were aligned at higher rather than lower levels, and that a follower was more likely to perceive higher levels of LMX quality when the focal person was more proactive than his/her leader, as compared with when the leader was more proactive than his/her follower. All of these findings remind us that we should focus on the more nuanced effects of congruence and incongruence effects when examining the effect of P–E fit at work.

### Practical Implications

Our findings also have several important practical implications. First, our findings suggest that, if an organization provides excessive supplies in work–family integration, employees may be hindered from benefiting from their own work–family integration preferences. Thus, it is crucial for employees to be aware of both their own work–family boundary preferences and organizational norms regarding work–family boundary management. With the increasing overlap between the home and family domains (Allen et al., 2014), organizations often encourage employees to continue to work after they go home (Madden and Jones, 2008). However, when employees perceive greater organizational supplies than their personal preferences, they may experience more stress and encounter more difficulties in maintaining their work and family balance. Therefore, it is important for organizations or managers to become aware of organizational norms on work–family boundary management and to endeavor to match these with their employees’ personal preferences.

Extant research has suggested that high levels of organizational supplies in work–family integration can have several detrimental effects on both work– and individual–related outcomes (Glavin and Schieman, 2012; Kossek et al., 2012). However, our findings indicate that these kinds of organizational norms may not always yield negative outcomes. In fact, the congruence effect we found suggests that, in a situation in which both personal preferences and organizational supplies are congruent at a high level, relatively high levels of work–family balance and positive family-related outcomes may still be obtained. However, higher congruence values of organizational supplies and personal preferences are not optimal. In such case, employees’ work–family balance is lower, compared to circumstances featuring a lower congruence in supplies and preferences. Managers should be careful in implementing high integration policies such as providing smartphones to employees and paying for supplemental work as these might decrease work–family balance by depleting finite resources (e.g., time, self–regulatory resources), especially when these approaches are approved and prevalent. Highly ambitious employees should also note that a willingness to engage in work-related tasks during off–job time may not be as advantageous as they think and may decrease their work–family balance, given the finite nature of the resources that are essential to work and family duties. Instead, they should consider reserving some time and energy with which to fulfill their family obligations, in order to obtain and maintain better work–family balance.

### Limitations and Future Research

Several limitations are associated with the present study. First, all the measures were evaluated in China, which may limit the generalizability of the results. In this cultural context, employees typically seek for some support for their own preferences. Therefore, the congruence effect would likely be stronger in China than elsewhere. Future research could address this limitation by comparing P–E fit in work–family integration across different cultures.

Second, the methodology of this study does not permit strong causal inferences. Although the data were collected at two time points, the cross-sectional design still constrains inferences of causality. Future studies could address this limitation by conducting their research with a more rigorous time-lagged design or an experimental design. Another potential problem could be that the data were self-reported, which may result in common method bias and inflated relationships among the focal variables. Future studies might rely on multiple sources of data. For instance, forthcoming research could assess workplace policies to represent the organizational supplies of individuals’ preferences for integration and link them to individual preferences to test the P–E fit effects.

Third, caution is advised regarding generalizing our results since the present study focused on married employees working 8-h day shifts, whereas some employees will have non–traditional work schedules, such as shift–workers and those holding part–time jobs. Future studies would attempt to replicate our findings among employees who work shift or in part–time roles.

The final limitation is that we regarded work–family balance as a global construct and measured it by evaluating one’s satisfaction with balancing work and family. Other researchers who supported the global construct of work–family balance have proposed that work–family balance refers not to one’s satisfaction with but to the effectiveness at balancing work and family. They defined work–family balance as the “accomplishment of role–related expectations that are negotiated and shared between an individual and his/her role–related partners in the work and family domains” (Grzywacz and Carlson, 2007, p. 458). Balance effectiveness may operate differently than balance satisfaction in predicting work– and family–related outcomes (Wayne et al., 2017). Therefore, future research would attempt to examine the effect of congruence/incongruence in work–family integration preference and organizational supplies of work–family integration on work–family balance and the effect of work–family balance on distal family–related outcomes from different definition and measurement of work–family balance (i.e., balance effectiveness).

## Data Availability

The datasets generated for this study are available on request to the corresponding author.

## Ethics Statement

This study was approved by the ethical committee of the North China University of Science and Technology. We introduced our research purpose, goals, and plans to each employee and asked their permission to participate in this research. Employees can withdraw from the study at any time without penalization. We obtained written informed consent from all participants before data collection.

## Author Contributions

All authors designed the study. PL and XW designed the questionnaires. XW collected the data. PL analyzed the results and wrote the manuscript. AL, XW, and LZ revised the manuscript.

## Conflict of Interest Statement

The authors declare that the research was conducted in the absence of any commercial or financial relationships that could be construed as a potential conflict of interest.

## References

[B1] Abendroth A. K. den Dulk L. (2011). Support for the work–life balance in Europe: the impact of state, workplace and family support on work–life balance satisfaction. *Work Emp. Soc.* 25 234–256. 10.1177/0950017011398892

[B2] Allen T. D. Cho E. Meier L. L. (2014). Work–family boundary dynamics. *Annu. Rev. Organ. Psychol. Organiz. Behav.* 1 99–121. 10.1146/annurev-orgpsych-031413-091330

[B3] Allen T. D. Herst D. E. L. Bruck C. S. Sutton M. (2000). Consequences associated with work-to-family conflict: a review and agenda for future research. *J. Occupat. Health Psychol.* 5 278–308. 10.1037/1076-8998.5.2.278 10784291

[B4] Aryee S. Srinivas E. S. Tan H. H. (2005). Rhythms of life: antecedents and outcomes of work–family balance in employed parents. *J. Appl. Psychol.* 90 132–146. 10.1037/0021-9010.90.1.132 15641894

[B5] Ashforth B. E. Kreiner G. E. Fugate M. (2000). All in a day’s work: boundaries and micro role transitions. *Acad. Manag. Rev.* 25 472–491. 10.5465/amr.2000.3363315

[B6] Atwater L. E. Ostroff C. Yammarino F. J. Fleenor J. W. (1998). Self–other agreement: does it really matter? *Pers. Psychol.* 51 577–598. 10.1111/j.1744-6570.1998.tb00252.x

[B7] Barber L. K. Taylor S. G. Burton J. P. Bailey S. F. (2017). A self-regulatory perspective of work-to-home undermining spillover/crossover: examining the roles of sleep and exercise. *J. Appl. Psychol.* 102 753–763. 10.1037/apl0000196 28150983

[B8] Beham B. Drobnič S. (2010). Satisfaction with work–family balance among german office workers. *J. Manag. Psychol.* 25 669–689. 10.1108/02683941011056987

[B9] Brislin R. W. (1980). “Translation and content analysis of oral and written material,” in *Handbook of Cross-Cultural Psychology, Vol. 2: Methodology*, eds Triandis H. C. Berry J. W. (Boston, MA: Allyn and Bacon), 349–444.

[B10] Buffardi L. C. Smith J. I. O’Brien A. S. Erdwins C. J. (1999). The impact of dependent care responsibility and gender on work attitudes. *J. Occupat. Health Psychol.* 4 356–367. 10.1037/1076-8998.4.4.356 10526840

[B11] Byron K. (2005). A meta-analytic review of work–family conflict and its antecedents. *J. Vocat. Behav.* 67 169–198. 10.1016/j.jvb.2004.08.009

[B12] Carlson D. S. Grzywacz J. G. Kacmar K. M. (2010). The relationship of schedule flexibility and outcomes via the work–family interface. *J. Manag. Psychol.* 25 330–355. 10.1108/02683941011035278

[B13] Carlson D. S. Kacmar K. M. Grzywacz J. G. Tepper B. Whitten D. (2013). Work–family balance and supervisor appraised citizenship behavior: the link of positive affect. *J. Behav. Appl. Manag.* 14 87–106. 10.1037/t03592-000

[B14] Casper W. J. DeHauw S. Wayne J. H. Greenhaus J. (2014). A review of the meaning and measurement of work–life balance. In *Proceedings of the 29th Annual Conference of the Society for Industrial–Organizational Psychology*, Honolulu, HI.

[B15] Chen Z. Powell G. N. Greenhaus J. H. (2009). Work-to-family conflict, positive spillover, and boundary management: a person–environment fit approach. *J. Vocat. Behav.* 74 82–93. 10.1016/j.jvb.2008.10.009

[B16] Clarke M. C. Koch L. C. Hill E. J. (2004). The work–family interface: differentiating balance and fit. *Fam. Consum. Sci. Res. J.* 33 121–140. 10.1177/1077727X04269610

[B17] Ďuranová L. Ohly S. (2015). *Persistent Work-Related Technology Use, Recovery and Well-Being Processes: Focus on Supplemental Work After Hours.* Berlin: Springer.

[B18] Edwards J. R. (1996). An examination of competing versions of the person–environment fit approach to stress. *Acad. Manag. J.* 39 292–339. 10.5465/256782

[B19] Edwards J. R. (2002). “Alternatives to difference scores: polynomial regression analysis and response surface methodology,” in *Advances in measurement and data analysis*, eds Drasgow F. Schmitt N. W. (San Francisco, CA: Jossey-Bass), 350–400.

[B20] Edwards J. R. (2008). Person–environment fit in organizations: an assessment of theoretical progress. *Acad. Manag. Ann.* 2 167–230. 10.5465/19416520802211503

[B21] Edwards J. R. Parry M. E. (1993). On the use of polynomial regression equations as an alternative to difference scores in organizational research. *Acad. Manag. J.* 36 1577–1613. 10.5465/256822

[B22] Edwards J. R. Rothbard N. P. (1999). Work and family stress and well-being: an examination of person–environment fit in the work and family domains. *Organ. Behav. Hum. Decision Process.* 77 85–129. 10.1006/obhd.1998.2813 10069942

[B23] Epstein N. B. Baldwin L. M. Bishop D. S. (1983). The McMaster family assessment device. *J. Mar. Fam. Ther.* 9 171–180. 10.1111/j.1752-0606.1983.tb01497.x

[B24] Ferguson M. Carlson D. Kacmar K. M. (2015). Flexing work boundaries: the spillover and crossover of workplace support. *Pers. Psychol.* 68 581–614. 10.1111/peps.12084

[B25] Frone M. R. (2003). “Work–family balance,” in *Handbook of Occupational Health Psychology*, eds Quick J. C. Tetrick L. E. (Washington, DC: American Psychological Association), 143–162.

[B26] Glavin P. Schieman S. (2012). Work–family role blurring and work–family conflict: the moderating influence of job resources and job demands. *Work Occupat.* 39 71–98. 10.1177/0730888411406295

[B27] Glenn N. D. (1990). Quantitative research on marital quality in the 1980s: a critical review. *J. Marriage Fam.* 52 818–831. 10.2307/353304

[B28] Grawitch M. J. Maloney P. W. Barber L. K. Mooshegian S. E. (2013). Examining the nomological network of satisfaction with work-life balance. *J. Occupat. Health Psychol.* 18 276–284. 10.1037/a0032754 23688250

[B29] Greenhaus J. H. Allen T. D. (2011). “Work–family balance: a review and extension of the literature,” in *Handbook of Occupational Health Psychology*, eds Quick J. C. Tetrick L. E. (Washington, DC: American Psychological Association), 165–183.

[B30] Grzywacz J. G. Carlson D. S. (2007). Conceptualizing work–family balance: implications for practice and research. *Adv. Dev. Hum. Res.* 9 455–471. 10.1177/1523422307305487

[B31] Guilford R. Bengtson V. (1979). Measuring marital satisfaction in three generations: positive and negative dimensions. *J. Marriage Fam.* 39 387–398. 10.2307/351705 19485674

[B32] Heider F. (1958). *Psychology of Interpersonal Relations.* New York, NY: Wiley.

[B33] Hughes E. L. Parkes K. R. (2007). Work hours and well-being: the roles of work-time control and work–family interference. *Work Stress* 21 264–278.

[B34] Jansen K. J. Kristof-Brown A. L. (2005). Marching to the beat of a different drummer: examining the impact of pacing congruence. *Organ. Behav. Hum. Decision Process.* 97 93–105. 10.1016/j.obhdp.2005.03.005

[B35] Jiang H. Wang L. Zhang Q. Liu D. X. Ding J. Lei Z. (2015). Family functioning, marital satisfaction and social support in hemodialysis patients and their spouses. *Stress Health* 31 166–174. 10.1002/smi.2541 24470353

[B36] Julien M. (2007). *Finding Solutions to Work-Life Conflict: Examining Models of Control Over Work-Life Interface.* Doctoral thesis, Kingston, ON, Queen’s University.

[B37] Kossek E. E. Ruderman M. N. Braddy P. W. Hannum K. M. (2012). Work–nonwork boundary management profiles: a person-centered approach. *J. Vocat. Behav.* 81 112–128. 10.1016/j.jvb.2012.04.003

[B38] Kreiner G. E. (2006). Consequences of work–home segmentation or integration: a person–environment fit perspective. *J. Organ. Behav.* 27 485–507. 10.1002/job.386

[B39] Liao Y. Yang Z. Wang M. Kwan H. K. (2016). Work–family effects of LMX: the moderating role of work–home segmentation preferences. *Leadership Q.* 27 671–683. 10.1016/j.leaqua.2016.03.003

[B40] Liu S. Gui D. Y. Zuo Y. (2019). Good slang or bad slang? Embedding internet slang in persuasive advertising. *Front. Psychol.* 10:1251. 10.3389/fpsyg.2019.01251 31231278 PMC6566129

[B41] Lu J.-F. Siu O.-L. Spector P. E. Shi K. (2009). Antecedents and outcomes of a fourfold taxonomy of work–family balance in Chinese employed parents. *J Occupat. Health Psychol.* 14 182–192. 10.1037/a0014115 19331479

[B42] Madden M. Jones J. (2008). *Networked Workers.* Washington, DC: Pew Internet & American Life Project.

[B43] McNall L. A. Scott L. D. Nicklin J. M. (2015). Do positive affectivity and boundary preferences matter for work–family enrichment? a study of human service workers. *J. Occupat. Health Psychol.* 20 93–104. 10.1037/a0038165 25347683

[B44] Miller I. W. Ryan C. E. Keitner G. I. Bishop D. S. Epstein N. B. (2000). The McMaster approach to families: theory, assessment, treatment and research. *J. Fam. Ther.* 22 168–189. 10.1111/1467-6427.00145

[B45] Muthén L. K. Muthén B. O. (2017). *Mplus User’s Guide* (8th edn). Los Angeles, CA: Muthén and Muthén.

[B46] Powell G. N. Greenhaus J. H. (2010). Sex, gender, and the work-to-family interface: exploring negative and positive interdependencies. *Acad Manag. J.* 53 513–534. 10.5465/amj.2010.51468647

[B47] Trent K. South S. J. (1992). Sociodemographic status, parental background, childhood family structure, and attitudes toward family formation. *J. Marriage Fam.* 54 427–439.

[B48] Valcour M. (2007). Work-based resources as moderators of the relationship between work hours and satisfaction with work–family balance. *J. Appl. Psychol.* 92 1512–1523. 10.1037/0021-9010.92.6.1512 18020793

[B49] Voydanoff P. (2005). Toward a conceptualization of perceived work–family fit and balance: a demands and resources approach. *J. Marriage Fam.* 67 822–836. 10.1111/j.1741-3737.2005.00178.x

[B50] Wang X. Li A. Liu P. Rao M. (2018). The relationship between psychological detachment and employee well-being: the mediating effect of self-discrepant time allocation at work. *Front. Psychol.* 9:2426. 10.3389/fpsyg.2018.02426 30618910 PMC6297841

[B51] Wayne J. H. Butts M. M. Casper W. J. Allen T. D. (2017). In search of balance: a conceptual and empirical integration of multiple meanings of work–family balance. *Pers. Psychol.* 70 167–210. 10.1111/peps.12132

[B52] Wilson K. S. Baumann H. M. Matta F. K. Ilies R. Kossek E. E. (2018). Misery loves company: an investigation of couples’ interrole conflict congruence. *Acad. Manag. J.* 61 715–737. 10.5465/amj.2016.0395

[B53] Yang L. Q. Che H. Spector P. E. (2008). Job stress and well-being: an examination from the view of person–environment fit. *J. Occupat. Organ. Psychol.* 81 567–587. 10.1348/096317907X243324

[B54] Zhang Z. Wang M. O. Shi J. (2012). Leader–follower congruence in proactive personality and work outcomes: the mediating role of leader–member exchange. *Acad. Manag. J.* 55 111–130. 10.5465/amj.2009.0865

